# Lack of evidence for trans-generational immune priming against the honey bee pathogen *Melissococcus plutonius*

**DOI:** 10.1371/journal.pone.0268142

**Published:** 2022-05-09

**Authors:** Florine Ory, Vincent Duchemin, Verena Kilchenmann, Jean-Daniel Charrière, Benjamin Dainat, Vincent Dietemann

**Affiliations:** 1 Swiss Bee Research Centre, Agroscope, Bern, Switzerland; 2 Department of Ecology and Evolution, University of Lausanne, Lausanne, Switzerland; Universitat Leipzig, GERMANY

## Abstract

Trans-generational immune priming involves the transfer of immunological experience, acquired by the parents after exposure to pathogens, to protect their progeny against infections by these pathogens. Such natural mechanisms could be exploited to prevent disease expression in economically important insects, such as the honey bee. This mechanism occurs when honey bee queens are exposed to the pathogenic bacterium *Paenibacillus larvae*. Here, we tested whether natural or experimental exposure to *Melissococcus plutonius*—another bacterium triggering a disease in honey bee larvae—reduced the susceptibility of the queen’s progeny to infection by this pathogen. Because the immunological response upon pathogen exposure can lead to fitness costs, we also determined whether experimental exposure of the queens affected them or their colony negatively. Neither natural nor experimental exposure induced protection in the honey bee larvae against the deleterious effects of *M*. *plutonius*. Our results provided no evidence for the occurrence of trans-generational immune priming upon exposure of the queen to *M*. *plutonius*. Whether this lack was due to confounding genetic resistance, to unsuitable exposure procedure or to the absence of trans-generational immune priming against this pathogen in honey bees remains to be determined.

## Introduction

As a managed pollinator of crops and producer of honey, the honey bee, *Apis mellifera*, has a high economic importance worldwide [1, 2]. In the last decade, the loss of managed honeybee colonies has increased in the northern hemisphere [3]. This increase threatens the pollination services provided by honey bees [4, 5]. The annual colony loss rates experienced by beekeepers range from 3% to up to 40% of their stock, depending on the area and year [6–8]. Such declines have been associated with several factors, such as the extensive use of pesticides in agriculture and diseases caused by parasites and pathogens, or with combinations of factors (e.g. [9–11]).

Among honey bee pathogens, the bacterium *Melissococcus plutonius* causes European foulbrood (EFB), a severe enteric disease that affects the larvae [12, 13]. The colour of infected larvae shift from pearly white—which is a characteristic for healthy individuals—to yellow-brown or greyish-black, and the larvae usually fail to complete pupation, which in the worst-case scenario, leads to colony death [14]. Adult workers contaminate themselves while removing larval cadavers. Although they are not susceptible to *M*. *plutonius*, they act as carriers of the bacterium and spread it within their colonies during brood feeding and nursing [15]. Furthermore, they can spread the pathogen between colonies and apiaries via robbing and drifting [16]. *M*. *plutonius* can easily spread at the regional scale, leading to severe outbreaks [17]. Recently, outbreaks have been reported in the Czech Republic, Finland, France, Greece, Holland, Italy, Norway, the United Kingdom and Switzerland [17]. In many countries, when detected, the disease must be reported to veterinary authorities; the affected apiaries are then subjected to strict sanitation measures, including restrictions on the movement of colonies, the destruction of symptomatic and weak colonies and thorough disinfection of beekeeping equipment [17]. No substitute medication exists for colonies affected by EFB in countries in which the use of antibiotics is forbidden. Antibiotics are not considered sustainable control methods because they do not kill the resistance forms of the bacteria, can lead to the occurrence of residues in honey and can lead to the possible emergence of resistance in their targets [14, 18].

Because of the lack of curative control methods and because of the high costs in terms of the time and resources required for sanitation measures, alternative solutions to control this disease are desirable. Honey bees are social insects and possess individual as well as social immune defences against pathogens [19, 20]. Because all workers in a colony are the progeny of a single individual (i.e. the queen), an immune mechanism driven by the queen and amenable to manipulation or programming could be exploited in a practical and economical manner to protect colonies from pathogens such as *M*. *plutonius*. Trans-generational immune priming (TGIP) is such a mechanism, in which the parental immunological experience is transferred to the progeny, leading to its protection against infections [21]. Commonly occurring in vertebrates [22], TGIP was also shown to occur in invertebrates [23] and has been evidenced in all coleopteran, crustacean, orthopteran and mollusc species investigated to date [21]. Social Hymenoptera express TGIP against diverse pathogens, such as fungi (in the ants *Formica fusca* and *Crematogaster scutellaris* [24, 25], viruses (in honey bees [26]) and bacteria (in bumble bees [27–30] and honey bees [31, 32]). TGIP led to a significant increase in survival of their brood when honey bee queens were exposed to the pathogenic bacterium *Paenibacillus larvae*, which causes American foulbrood [31].

Here, we tested the hypothesis that the offspring of honey bee queens exposed to *M*. *plutonius* better survive infection by this bacteria than offspring of non-exposed queens. Our goal was to provide a proof-of-concept towards the development of a TGIP-based preventive treatment against EFB. For this, we exposed queens to *M*. *plutonius* orally once or twice with two doses of bacteria. The survival of the larvae reared *in vitro* were measured before the queens were exposed to the bacteria to determine the baseline mortality when infected with *M*. *plutonius*. We measured the larval survival a second time, after exposure of the queen, to determine the degree of protection achieved. To consider potential TGIP-associated fitness costs to the queen and colony [33], which would jeopardise the economic sustainability of exploiting TGIP, we evaluated the impact of the procedure by measuring the survival and brood production of the queen *in vivo*. Because successful triggering of TGIP can depend on the experimental conditions of pathogen exposure [21, 26], we also tested whether TGIP occurs naturally by comparing the survival to *M*. *plutonius* infection of brood produced by queens of EFB-diseased field colonies and of healthy colonies.

## Material and methods

### Honey bee colonies

The honey bee colonies used were kept at the apiary of the Swiss Bee Research Centre in Liebefeld, Switzerland, under local good beekeeping practices [34], including treatments against *Varroa destructor* with organic acids once the inoculations assays were completed. Yearly PCR screening for *M*. *plutonius* (SM 1 in S1 File) was performed to ensure that the colonies used were free of natural infection [35, 36]. For the experiments, colonies were kept in small-sized (Mini Plus) hives. In the experimental exposure assay (2019–2020), colonies were headed by locally mated queens. The ages of these queens ranged between less than a year (born during the experimental year) to 3 years old (S1 Table). The queens were unrelated to ensure broad genetic variability among the test units.

In the natural exposure assay (2021), ten queens were obtained from symptomatic colonies of four apiaries in the Canton of Bern, Switzerland. To confirm their infection by *M*. *plutonius*, ten grams of workers were collected from these colonies and analysed using PCR (SM 1 in S1 File). Because symptomatic colonies have to be destroyed by law [17], the queens were removed from their colony of origin and introduced into 10 previously dequeened healthy colonies to allow for experimentation. A total of 20 healthy colonies were used, ten headed by naturally exposed queens, and ten headed by non-exposed queens (S2 Table).

### Bacterial cultivation and inoculum preparation

*M*. *plutonius* strains CH49.3 and CH45.1 were used as infectious agents of EFB for larval inoculation and queen exposure in the test for experimental triggers of TGIP and in the test for natural occurrence of TGIP, respectively. CH49.3 was chosen for its high virulence [17] to determine whether TGIP could protect from highly damaging strains. CH45.1 was chosen for its medium virulence [17], which may, on average, be more representative of infections in the field. The bacteria were both isolated from EFB-diseased colonies in Switzerland and stored at -80°C in 15% glycerol solution until use. In contrast to the study conducted by Hernández López et al. (2014) [31], viable and not inactivated bacteria were used for exposure, in case inactivation hindered the putative priming mechanism. The bacterium was cultivated on solid basal medium containing 10 g/l yeast extract, 10 g/l glucose, 10 g/l starch, 20 g/l agar, 0.25 g/l L-cysteine and 1 M KH_2_PO_4_ in distilled water, adjusted to pH 6.7 using 2.5 M KOH and autoclaved at 115°C for 15 minutes [37]. After four days of incubation at 36°C under anaerobic conditions (hermetic box with anaerobic generator sachet, GENbox anaer, bioMérieux and anaerobic indicator), the bacterial colonies were suspended in liquid basal medium (same recipe as described above, but without agar and with 10 g/l saccharose instead of starch) and stored at 4°C for four days. The bacterial concentration in the medium was determined with ten-fold serial dilutions and by counting the colony-forming units (CFUs) grown on Petri dishes, under the previously described cultivation conditions. Bacterial inocula were prepared immediately before larval inoculation and queen exposure; the desired bacterial concentration (see below) was adjusted in sterile saline suspension buffer (0.9% NaCl).

Sugar-royal jelly mixes and sugar compositions of the larval diet and queen food, are known to have antibacterial properties [38, 39]. To evaluate the extent of this bactericidal effect, the amount of viable bacteria in the inocula given to larvae and queens was quantified. The bacterial inocula were plated after each experimental inoculation session. For this, 50 μl of bacterial inoculum (i.e. of bacteria-containing diet) at three dilutions (1:10^2^, 1:10^3^, 1:10^4^) as well as control food (dilution 1:10^2^ only) were plated within three hours post-inoculation on solid medium plates [40]. CFUs were counted after four days of anaerobic incubation at 36°C and are given in CFUs per individual.

### *In vitro* larval inoculation assays

**Fig 1:**
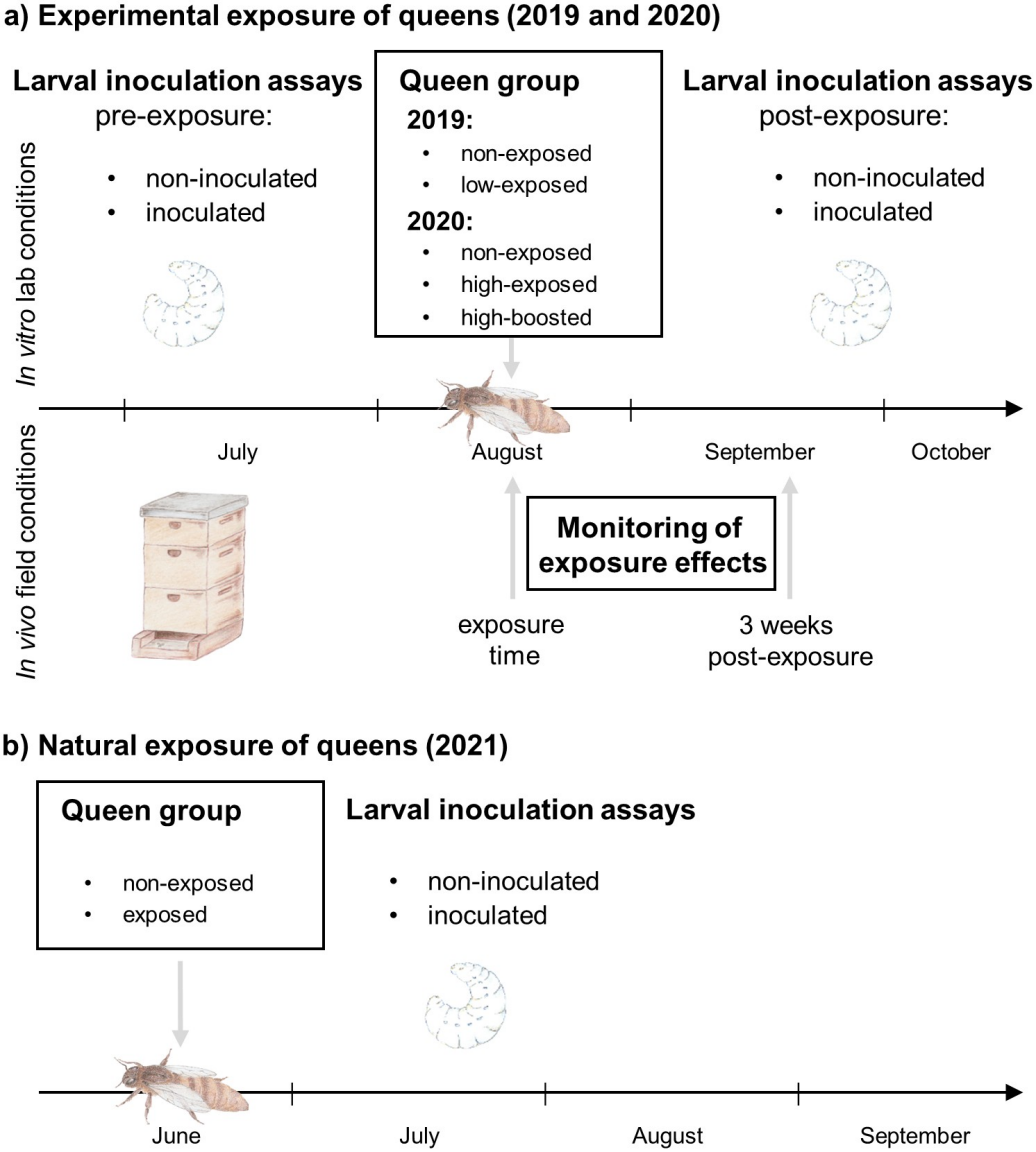
Experimental plan of the study. a) Test for experimental trigger of TGIP, and b) test for natural occurrence of TGIP.

To test whether TGIP can be triggered experimentally, honey bee larvae originating from healthy colonies were reared *in vitro* before the queen was exposed to the bacterium to assess the basal mortality induced by *M*. *plutonius* inoculation. Mortality was measured again after the exposure of the queen to assess the protective effect of this exposure on the brood (Fig 1a). For *in vitro* rearing, numerous same-aged first instar larvae were obtained by caging the queens on empty combs using excluder cages [41]. Excluder cages restrict the queens’ but not the workers’ movements to the caged comb. Thus, the queens were coerced to lay eggs on the caged combs, while receiving care from workers. After a 24-hour period, the queens were removed from the cages. Three days post removal, the freshly hatched larvae were picked using a paintbrush (size 2/0) and grafted into plastic queen starter cells (Nicotplast^™^). Both the paintbrush and plastic cups had previously been sterilised for at least 2 hours in 70% ethanol. Starter cells were placed into 48-well suspension culture plates, on top of pieces of cotton dental rolls that were soaked with 500 μl of 15.5% glycerol in 0.4% methyl benzethonium chloride to prevent contamination by unwanted microorganisms. *In vitro* larval rearing was performed as described by Aupinel et al. (2005) [42]. Briefly, for the first six days, the plates were placed in a Perspex box, in which a saturated potassium sulphate (K_2_SO_4_) solution maintained a relative humidity of 95% required to prevent larval dehydration. At day seven, excess food that was not consumed by the larvae was removed from the starter cells, which were then transferred into new sterile 48-well culture plates and placed into a second box in which a saturated sodium chloride solution maintained a relative humidity of 80% to allow metamorphosis. Both the boxes were placed in an incubator at 34.5°C. The larvae were fed according to the following protocol: on the first day, the grafted larvae received 10 μl of diet A (S3 Table). Within two hours of grafting, the inoculated larvae received an additional 10 μl of diet A containing 2 × 10^7^ CFU ml^-1^ (i.e. 2×10^5^ CFUs per larva) of *M*. *plutonius* bacteria (diet A: bacterial suspension, w:v, 9:1). Non-inoculated larvae received 10 μl of control diet A (diet A: suspension buffer, w:v, 9:1). On day three, all larvae were fed 20 μl of diet B (S3 Table). On days four, five and six, the larvae received 30, 40, and 50 μl of diet C, respectively (S3 Table). Sugars (D-glucose and D-fructose) and yeast extract were dissolved in milliQ water, and the solutions were filtered through a 0.2 μm mesh cellulose acetate filter and combined with royal jelly.

Royal jelly for the experimental trigger of TGIP was harvested from healthy queen-less colonies at the Swiss Bee Research Centre, and organic royal jelly was obtained from a commercial source of hive products (Werner Seip, Germany) to test the natural occurrence of TGIP. The jellies were stored at -25°C in a sterile environment until use. Honey bee larvae received a single *M*. *plutonius* inoculation on the day of grafting, after which, the inoculated and non-inoculated individuals were handled in the same manner. Brood survival was monitored from day three until the completion of development (i.e. until the imaginal stage). The status (dead or alive) of larvae was recorded every 24 hours until complete metamorphosis (day 13) through observation under a binocular microscope. An individual was considered to be dead when neither movement due to respiration nor reaction to a mechanical stimulus applied using a thin sterile rod were detected. The dead individuals were removed from the plates. After metamorphosis, the pupae are mostly immobile, and observing respiration becomes difficult; thus, the stage of the dead individuals was determined at the end of the experiment, when live imagoes emerged. The mortality of the non-inoculated brood was evaluated to assess the effect of *in vitro* rearing on mortality. A rearing series was included in the dataset when the mortality of the non-inoculated control individuals was below 25% [43]. In 2020, this threshold was increased to 35% because of the consistently higher control brood mortality of one queen. For this experiment, a total number of 2’148 larvae from 10 queens were reared in 2019, and 2’136 from nine queens in 2020. The sample sizes of each group and replicates (i.e. grafting series) are listed in S1 Table. Replicates were performed before and after exposure of the queen at intervals of seven days over a period of at least three weeks to control for potential environmental effects on brood survival.

Once the pre-exposure larval inoculation assays were completed, queens were collected, exposed to *M*. *plutonius* (exposed group) or sham-treated (non-exposed group), and returned to their colonies of origin (Fig 1a). The post-exposure larval inoculation assays started three days after the exposure of the queen.

To test whether TGIP occurs naturally, honey bee larvae produced by queens from EFB-diseased colonies (exposed) and healthy colonies (non-exposed) were reared and inoculated (Fig 1b) in the same manner as that described above, except that the *M*. *plutonius* strain CH45.1 was used instead of CH49.3. For this experiment, a total of 1,718 larvae from 20 queens were reared in 2021. The sample sizes of each group and replicates are listed in S2 Table.

### Experimental exposure of the queen to *M*. *plutonius*

Queens were exposed to *M*. *plutonius* by oral inoculation. In 2019, four queens were randomly assigned to receive control food (non-exposed group) and six to receive food containing a low dose of 5×10^5^ CFUs of *M*. *plutonius* (low-exposed group). In 2020, three queens were assigned to receive control food (non-exposed group) and four to receive food containing a high dose of 1×10^7^ CFUs of *M*. *plutonius* (high-exposed group). Two doses (low and high) were used to determine whether the amount of bacteria in the inoculum affected the degree of protection of the progeny. In addition, two queens exposed to a low dose in 2019 were exposed again in 2020 (hereafter designated as ‘boosted’) to determine whether two inoculations increased the protection of their offspring. These queens were exposed for a second time with a high dose of 1×10^7^ CFUs of *M*. *plutonius* (Fig 1a). To avoid an unbalanced sample size between the control and treatment groups because of a higher potential probability of queen mortality after bacterial exposure, more queens were allocated to the exposed group. The colonies hosting test queens were regularly checked for the absence of EFB symptoms by visual inspection.

In contrast to the previous experiments in which queens were primed via injection in the hemocoel [31], the oral route was chosen for exposure because it stimulates the natural exposition route of honey bees to *M*. *plutonius* and because injection may present a higher risk of queen death compared to the oral route. For exposure, each queen was removed from its colony and confined in a Butler cage (Nicotplast^™^) supplied with candy (APIfonda) and seven workers. The cages were transported to the lab where the queens were transferred to a new Butler cage without any food or workers for a 10–20 min starvation period to increase their willingness to consume the inoculum. During this time, the queens were kept at room temperature. After this starvation period, the queens were fed using a sterile pipette tip. Exposed queens received 10 μl of a sugar diet (milliQ water:sugar, w:w, 1:1) containing *M*. *plutonius* suspended in sterile saline buffer (sugar diet/bacterial suspension, w:v, 9:1). Non-exposed queens received 10 μl of a sugar diet containing sterile saline buffer (sugar diet/suspension buffer, w:v, 9:1). The sugar diet was sterilised by 0.2 μm filtration prior to addition of the bacteria or sterile saline to avoid autoclaving, which leads to the formation of hydroxymethylfurfural (HMF), a sugar-derived molecule, which is highly toxic for honey bee workers [44]. The queens consumed the entirety of the inoculum provided within one to three minutes. The procedure of exposure lasted less than one hour to minimise stress to the queen and the risk of rejection by the workers. Immediately after food consumption, each Butler cage enclosing the queens was sealed with candy and returned to the colony. The queens were released from the cages by the workers when they consumed all the candy. The time needed for queen release allowed workers to readapt to her presence and increased the success of reintroduction.

### Monitoring the effect of exposure on queen fecundity

The effect of exposing queens to *M*. *plutonius* on their acceptance by workers was controlled the next day. The queens were monitored regularly for at least one month to verify if they survived the exposure. To evaluate the effect of queen exposure on their fecundity, their brood production was evaluated in their respective colonies at the time of queen exposure, which reflected their baseline fecundity, and three weeks after the exposure (Fig 1a). Brood production was measured by quantifying the comb surface area occupied by open and sealed brood following the Liebefeld method [45, 46]. For this, the colonies were opened, and the surface of the comb occupied by the brood on both sides of each frame was estimated. The proportional change in the brood surface between exposure time and three weeks later was used as a proxy for the change in queen fecundity. This change was calculated as follows:

$$\Delta BS=-\left(1-\frac{{BS}_{exposure}}{{BS}_{+3weeks}}\right)$$

where Δ*BS* is the proportional change in brood surface area, *BS*_exposure_ is the brood surface area at exposure time and *BS*_+3weeks_ is the brood surface area three weeks after exposure.

### Statistical analyses

The effect of exposure of the queen on brood survival was analysed separately for each year because queens were exposed to low bacterial doses in 2019 and high bacterial doses in 2020 and were naturally exposed in 2021. Cox mixed-effects models fitted by maximum likelihood [47] in R version 4.1.0 were used for the experimental trigger of TGIP and Version 4.0.2 for natural triggering [48]. Ties in death times were handled using the Breslow method. Brood survival was compared before and after experimental exposure of each queen as well as between exposed and non-exposed queens after exposure and between the inoculated and non-inoculated brood. In 2019, the survival of inoculated and non-inoculated brood pre- and post-exposure of their queens (i.e. non-exposed, low-exposed groups) was compared, for a total of eight categories (two categories of larval treatment × two periods × two groups of queens). In 2020, 12 categories were compared because the group of boosted queens was added. The comparisons were performed by modelling the interaction between the fixed effect ‘queen group’ (non-exposed, exposed, and boosted) in the pre- and post-exposure period and the ‘larval inoculation’ status (inoculated and non-inoculated). In 2021, the survival of brood produced by queens from EFB-diseased colonies (N = 10) was compared with that of brood produced by healthy queens (N = 10). The factors ‘queen group’ (i.e. naturally exposed vs. non-exposed) and ‘larval treatment’ (i.e. inoculated and non-inoculated) were considered as fixed effects, and their interactions were modelled. To consider any seasonal and individual effects (i.e. age and genetics of queens), ‘replicate’ and ‘queen identity’ were included as random factors, with the former nested in the latter. Pairwise comparisons were performed to determine the effects of the exposure status of the queen on brood survival. Tests were performed on the log scale, using the Tukey method for *p*-value adjustment after multiple testing.

To investigate the effect of queen exposure on brood production, proportional changes in the brood surface over three weeks were analysed according to the queen treatment through non-parametric tests. The Mann-Whitney test was used for the year 2019 (two groups of queens: low-exposed vs. non-exposed), and the pairwise Kruskal-Wallis test with Bonferroni-Holm p-value adjustment method was used for the year 2020 (three groups of queens: non-exposed vs. high-exposed vs. high-boosted). Kaplan-Meier survival curves and dotplots were created using the ‘ggplot2’ R package [49] and safe colourblind palette [50].

## Results

### Effect of experimental queen exposure on brood survival

None of the colonies in which queens were exposed to *M*. *plutonius* developed EFB symptoms.

**Fig 2:**
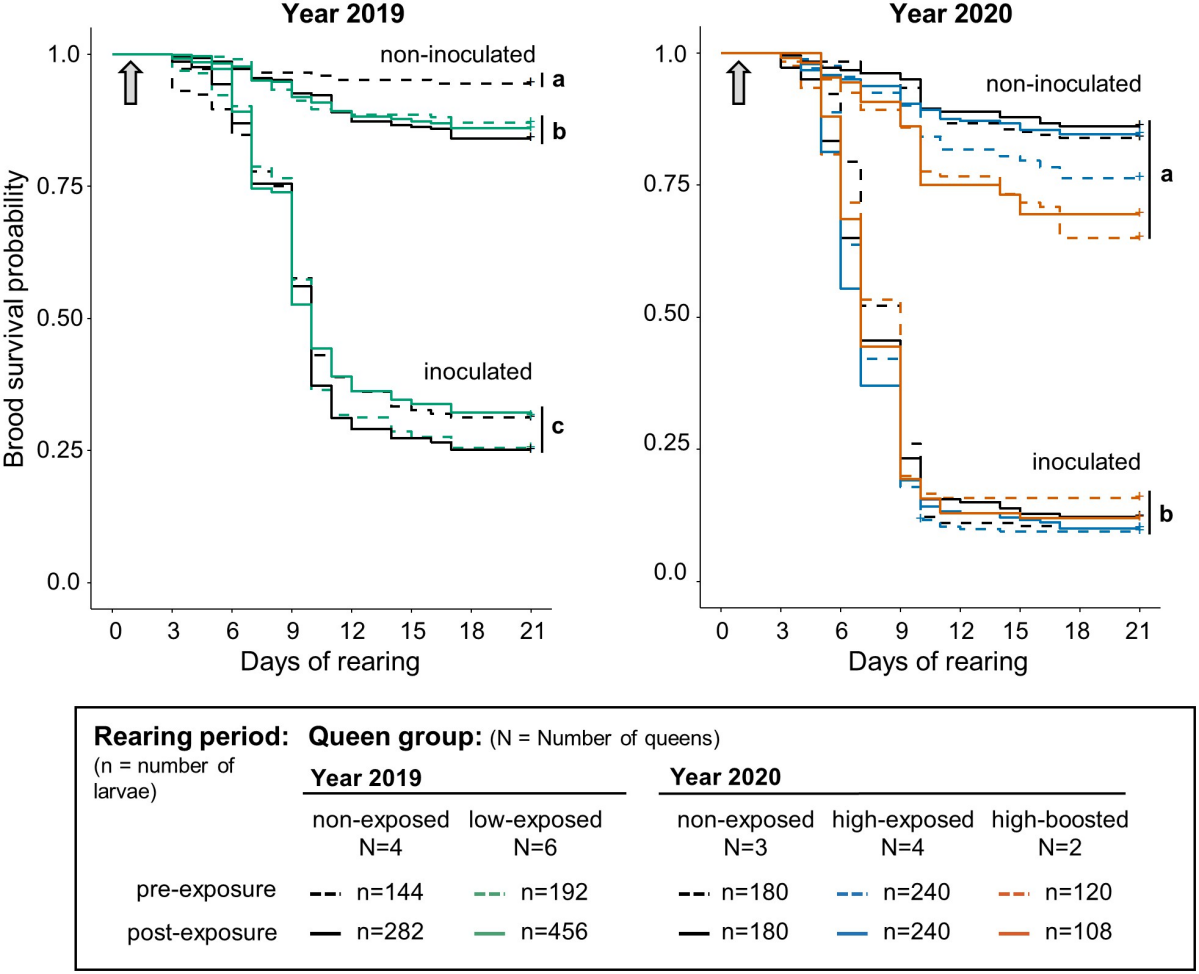
Brood survival probabilities when queens were experimentally exposed or not exposed to *Melissococcus plutonius*. Survival profiles of brood reared *in vitro* experimentally inoculated with *M*. *plutonius* on the day of grafting or non-inoculated during the years 2019 and 2020. Survival profiles of brood are displayed according to their queen’s exposure status (non-exposed, exposed, or boosted) and the rearing period (pre- and post-exposure). Grey arrows represent the day of larval exposure to *M*. *plutonius* or to non-inoculated diets. Different letters indicate groups of treatments that differed significantly (Cox mixed-effects model, *p* < 0.05). Survival curves grouped by vertical bars were not significantly different (Cox mixed-effects model, *p* > 0.05).

From the 2×10^5^ CFUs mixed with larval diet A, an average of 2.9×10^4^ to 4.4×10^4^ CFUs were recovered in the larval inocula (SM 2 in S1 File). For both 2019 and 2020, these inocula led to a significant reduction in larval survival as compared to the non-inoculated controls (Fig 2, SM 3A, 3C_1-4_, 4A, 4C_1-6_ in S1 File). Regarding the queen inocula, the numbers of *M*. *plutonius* CFUs quantified from the low and high doses contained fewer viable bacteria than initially intended, with 1.4×10^5^ and 6.9×10^6^ CFUs per individual, instead of 5×10^5^ and 1×10^7^, respectively (SM 2 in S1 File).

A paired comparison between queen groups in both years showed a single significant difference between non-exposed queens before and after exposure in 2019 (Fig 2, SM 3B, 3C_5-9_, 4B, 4C_7-18_ in S1 File). Brood survival probability was neither affected by queen exposure to a low dose in 2019 (Fig 2, SM 3C_9-11_ in S1 File) nor by exposure to a high dose in 2020 (Fig 2, SM 4C_22-23_ in S1 File). This was also the case after the queens were boosted with a second high dose of *M*. *plutonius* (Fig 2, SM 4C_24_ in S1 File).

### Effect of queen exposure on her survival and fecundity

**Fig 3:**
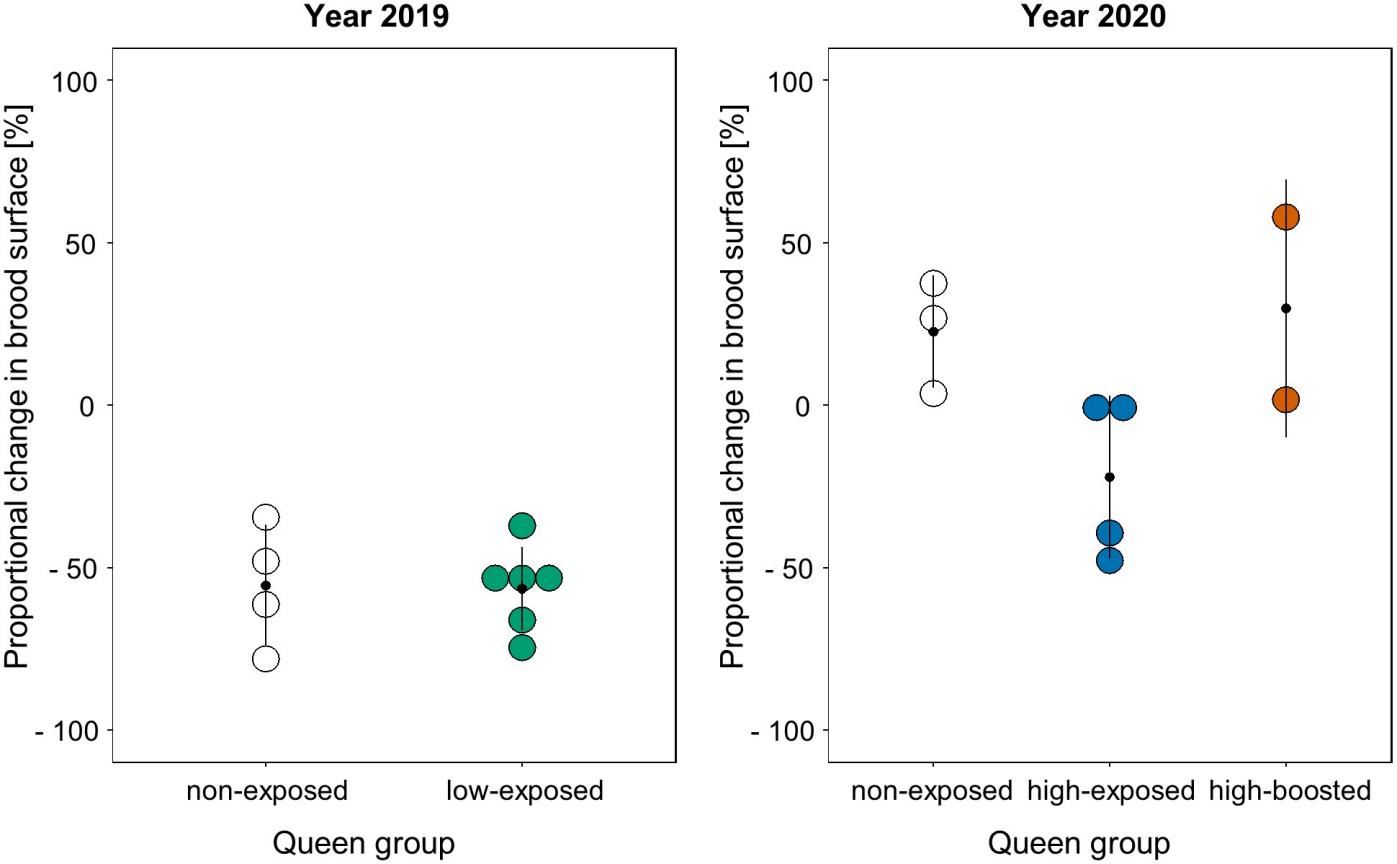
Brood production in colonies headed by queens experimentally exposed to *Melissococcus plutonius*. Proportional changes in brood surface area observed in colonies between the time of queen exposure and three weeks post-exposure in 2019 and 2020. Black dots and bars represent the mean and standard deviation of each group, respectively. Circles represent the individual data points.

The exposure method by feeding was well tolerated by the queens because they were all reaccepted by workers upon reintroduction into their colony and all survived for at least a month after exposure. In 2019, three weeks after queen exposure, the brood surface observed in the colonies was on average 56% smaller than that observed at exposure, without significant differences in the brood surface reduction of low-exposed queens compared to non-exposed queens (Fig 3, Wilcoxon rank sum test, W = 13, *p* = 0.92). In 2020, the brood surface was on average 4.3% higher than that at exposure, without significant differences related to queen treatment (Fig 3, Pairwise Wilcoxon rank sum test, Bonferroni-Holm adjusted, non-exposed vs. high-exposed, *p* = 0.17; non-exposed vs. high-boosted, *p* = 1.00; high-exposed vs. high-boosted, *p* = 0.80).

### Effect of natural queen exposure on brood survival

**Fig 4:**
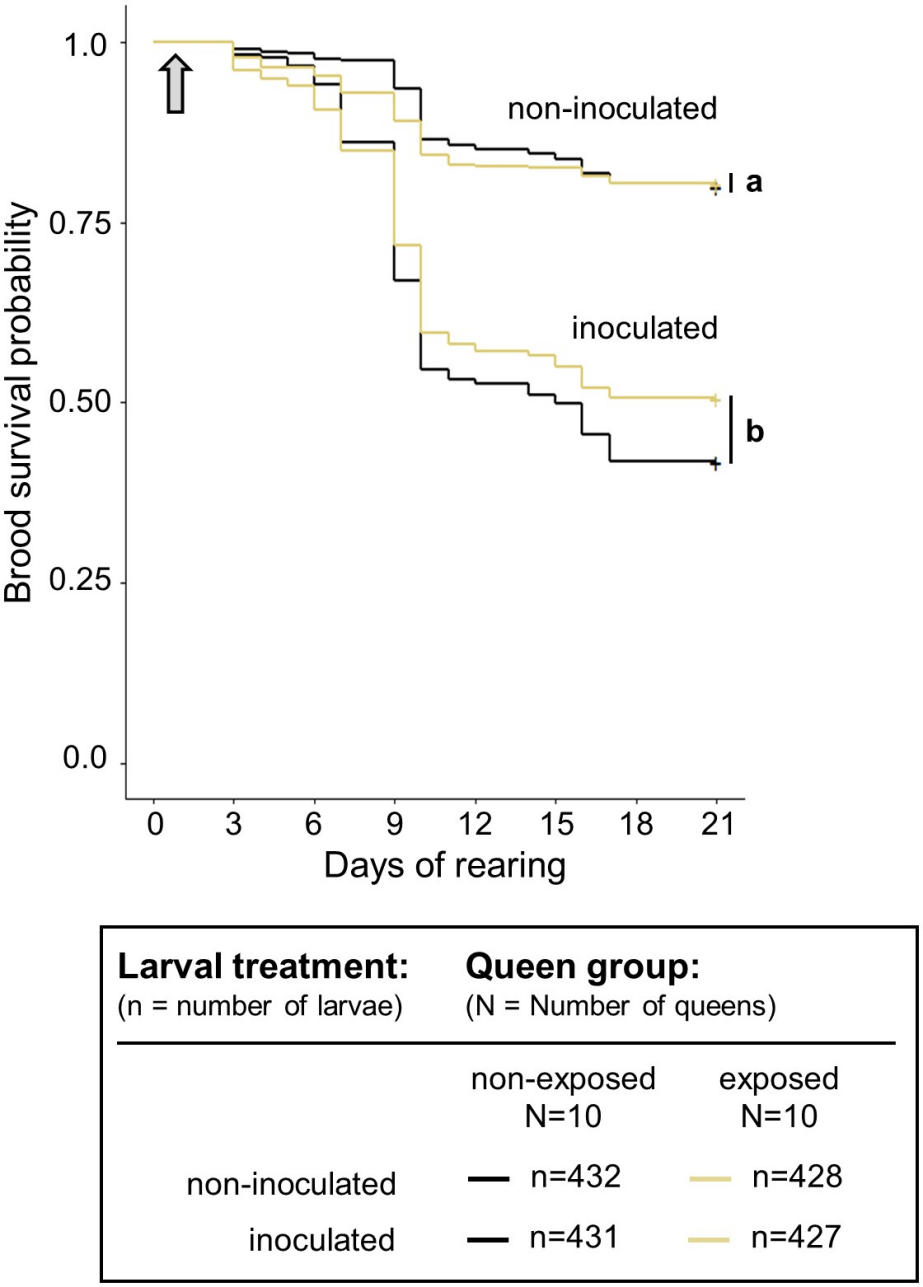
Survival probabilities of the brood from queens non-exposed or naturally exposed to EFB. Survival profiles of *in vitro* reared brood experimentally inoculated with *Melissococcus plutonius* on the day of grafting and the non-inoculated brood. Survival profiles of brood are displayed according to their queen’s exposure status (non-exposed and naturally exposed). Grey arrows represent the day of feeding with diet inoculated with *M*. *plutonius* or a non-inoculated diet. Different letters indicate treatments that differed significantly (p < 0.05) based on the Cox mixed-effects model. Survival curves grouped by a vertical bar on the right are not significantly different as per the Cox mixed-effects model (p > 0.05).

Positive signals or trace signals of *M*. *plutonius* presence were detected in all 10 EFB-diseased colonies (SM 1 in S1 File). Inoculation with *M*. *plutonius* had a significantly negative effect on the larval survival, but no significant differences could be detected between survival of brood produced by queens from healthy colonies and of brood produced by queens from diseased colonies (Fig 4, Table 1).

**Table 1:** Cox mixed-effects model of survival of inoculated and non-inoculated larvae produced by non-exposed queens from healthy colonies and by *Melissococcus plutonius* exposed queens from EFB-diseased colonies.

Fixed coefficients	coef	exp(coef)	se(coef)	z	p
larva_treatment1	1.40364925	4.0700254	0.1258558	11.15	0.00
queen_group1	-0.06399901	0.9380059	0.2399217	-0.27	0.79
larva_treatment1:queen_group1	-0.13118023	0.8770597	0.1806615	-0.73	0.47

## Discussion

We aimed to determine whether trans-generational immune priming against *M*. *plutonius* could be triggered experimentally and whether it occurs naturally. Exposing queens experimentally to *M*. *plutonius* by feeding them with viable bacteria was well tolerated over the short-term but did not protect their offspring from infection with the same pathogenic strain. Survival of brood produced by queens from healthy colonies or by queens collected from EFB-diseased colonies did not differ significantly when infected by *M*. *plutonius*.

### Effect of experimental exposure to *M*. *plutonius* on queen survival and fecundity

Colonies in which queens experimentally exposed to *M*. *plutonius* were introduced did not develop EFB symptoms until the following spring. All experimental exposure conditions (low and high dose and boost very close to those intended, SM 2 in S1 File) were well tolerated by the queens, as shown by their good acceptance by workers upon reintroduction in their colonies and by their survival over at least a month. However, the long-term effects of queen exposure should be investigated in more detail because a higher proportion (4/6) of exposed queens died within a year compared to non-exposed queens (0/4).

The changes in the brood production observed in the 2019 colonies headed by exposed queens were not significantly different from those in colonies headed by non-exposed queens (Fig 3). This indicates that the changes were not caused by the exposure but by the typical seasonal variations in brood production [51] or by the differences in beekeeping management between the years. The queen’s fecundity was not affected by the exposure at the tested doses. However, parameters other than brood surface areas should be considered to assess the effect of exposure on the queens’ physiology and colony vitality and fitness in more detail, especially because immunisation costs have been demonstrated in other social Hymenoptera [33]. Such parameters could be caused by the production of sexuals, queen longevity over several years or colony productivity.

### Effect of experimental queen exposure to *M*. *plutonius* on brood protection

Brood survival did not significantly differ within the queen groups before and after their exposure to *M*. *plutonius*, indicating that our assay was well randomised. The survival of uninfected brood only differed significantly before and after sham treatment in the non-exposed queens in 2019 (SM 3 in S1 File), an effect that we cannot explain, but which does not affect our conclusions regarding the occurrence of TGIP. The variance in the random factors of queen identity and replicate tended towards zero, showing that individual or seasonal effects did not largely influence brood survival.

Feeding larvae with viable *M*. *plutonius* cells significantly decreased their survival as compared to the non-inoculated brood (Fig 2), in line with the results of previous studies and showing successful infection (e.g. [17]). This decrease was of the same extent irrespective of queen treatment. Neither a single exposure at low or high dose nor a second exposure one year later with a high dose of *M*. *plutonius* had a significant or biologically relevant impact on brood survival (Fig 2). Our results differ from those obtained after exposing queens to *P*. *larvae* by Hernández López et al. (2014), who showed a protective effect, with a reduction of 26% in brood mortality after priming [31]. This discrepancy could be due to methodological differences. The queens used by Hernández López et al. (2014) were primed by injection of heat-killed bacteria into the haemocoel. Inactivation and a non-natural infection route, in this case, did not prevent TGIP [31]. The use of viable bacteria in our experiment could have led to the prevention of immunisation of queens by the live pathogen [52]. Although *M*. *plutonius* is not known to affect adult honey bees negatively [14, 53], no detailed study on the pathogen’s effects on the physiological processes in adults is available to date. TGIP could also be a dose-dependent mechanism, and our inocula could have contained an insufficient number of *M*. *plutonius* bacteria to trigger TGIP. Although the bactericidal effect of sugars and royal jelly in the diet decreased this number, it remained in the same order of magnitude or one order less than the dose intended, respectively (SM 2 in S1 File). Our doses were in the same order of magnitude or one order higher than those used by Hernández López et al. (2014) [31]. However, the ratio between minimal dose to trigger disease in the progeny and queen inoculum was two to three orders of magnitude higher in their experiment [31] compared to that in ours. Using higher inoculum doses of *M*. *plutonius* to potentially trigger TGIP could also have impacted queen health negatively, which would be counterproductive.

The lack of progeny protection might also be due to the oral delivery pathway of the inoculum. The bacteria transited via the digestive tract of *A*. *mellifera* queens, where they could be partly or totally fragmented [32]. To sustain TGIP, these fragments should transgress the epithelial gut barrier into the haemolymph and be transferred into ovaries by vitellogenin-bound transport. Vitellogenin-bound pathogen fragments then reach the oocytes [32, 54]. It is possible that this transit led to the dilution or destruction of the pathogenic cue from the gut to the egg, especially because the first step involves crossing the gut epithelium, which is a major immune barrier [19, 55]. If TGIP by *M*. *plutonius* is triggered by pathogenic cues, a single or double bacterial administration to the queen might not have been sufficient to induce immunisation and TGIP, and TGIP by *M*. *plutonius* could require a chronic exposure to the pathogen.

A difference in the expression of TGIP according to exposure route was also observed when honey bee queens were orally exposed to deformed wing virus or when injected with this virus [26]. This study suggested an interaction between the host genetics or epigenetics factors and the route of exposure in determining TGIP expression [26]. Such interactions could explain why other studies failed to orally induce TGIP in honey bee against the same inactivated virus [56]. Such contrasting results are not specific to honey bees and have been reported for Diptera and Lepidoptera. TGIP was not observed in all combinations of dipteran or lepidopteran host and pathogen species [21].

TGIP expression thus may depend on the pathogen used for priming, host genetics, epigenetic factors or methodological issues such as the exposure procedure (e.g. [21, 26]). Designing experiments to test the role of these factors and standardising experimental approaches are desirable to improve our understanding of TGIP mechanisms and their triggers.

### Effect of natural queen exposure to *M*. *plutonius* on brood protection

Methodological hindrance to the triggering of TGIP can be excluded when investigating the natural occurrence of this defence mechanism. We observed that the brood produced by queens likely exposed to *M*. *plutonius* (SM 1 in S1 File) naturally in EFB-diseased colonies did not survive infection by *M*. *plutonius* better than the brood produced by queens from healthy colonies. Although this result suggests the absence of TGIP against this pathogen, we cannot exclude the confounding effects of genetic resistance. Given the origin of the healthy colonies in a single population, their degree of resistance against *M*. *plutonius* may have obscured the benefit of TGIP in the colonies headed by queens of diseased colonies. If this were true, the benefits of TGIP would not be higher than those of genetic resistance to the pathogen in all populations. Alternatively, even though the bacteria was detected in their colonies (SM 1 in S1 File), queens could have escaped exposure to the pathogen via social distancing mechanisms [57].

## Conclusions

In the present study, we tested whether it was possible to induce TGIP in honey bees to decrease mortality of larvae infected with the pathogenic bacterium *M*. *plutonius*. TGIP could be exploited to protect colonies against EFB. Feeding queens with viable *M*. *plutonius* bacteria has not proven to be effective in increasing the survival of infected offspring. Our choice of exposure conditions by single feeding (rather than injection) that could be easily performed by beekeepers for colony health management and of a viable bacterium might have prevented immunisation. Despite the lack of evidence that natural exposure to the pathogen triggered TGIP, we do not exclude the fact that injection or feeding of deactivated *M*. *plutonius* bacteria or a chronic exposure of queens to low *M*. *plutonius* doses would stimulate immunisation. However, injections or chronic applications are more difficult or costly to implement by beekeepers themselves, but if conclusive, could be made available through commercialisation of treated queens. The priming procedure with *M*. *plutonius* thus requires optimisation to potentially reach a biologically relevant effect in terms of offspring survival while ensuring that the costs to queen and colony health and fitness remain minimal. Optimising the immunisation conditions and its transfer to the progeny would be facilitated by a deeper knowledge of the proximal mechanisms underlying TGIP in honey bees. The range of pathogens triggering this mechanism should also be determined to assess how widely this natural mechanism could be exploited to improve honey bee health in regions with high disease prevalence.

## Supporting information

S1 Table Queen age and number of larvae per queen per group and replicate used in the pre- and post-experimental exposure inoculation assays. (DOCX)

S2 Table Number of larvae per queen collected from EFB-diseased colonies (i.e. naturally exposed to *M*. *plutonius*) or from healthy colonies (i.e. non-exposed to *M*. *plutonius*). (DOCX)

S3 Table Composition of the diets used for larval rearing, according to feeding day. (DOCX)

S1 File (DOCX)
