# Supplementary material for: Lack of evidence for trans-generational immune priming against the honey bee pathogen Melissococcus plutonius
Source: PLoS One. 2022 May 9;17(5):e0268142. doi: 10.1371/journal.pone.0268142 (PMC9084521; doi:10.1371/journal.pone.0268142)
Supplement: S1 Table — (DOCX) [file pone.0268142.s001.docx]

**S1 Table. Queen age and number of larvae per queen per group and replicate used in the pre- and post-experimental exposure inoculation assays.**

|  |  |  |  | **Rearing period for inoculation assay**  **(number of larvae per replicate)** | |
| --- | --- | --- | --- | --- | --- |
| **Year** | **Queen** | **Year of birth** | **Queen group** | **Pre-exposure** | **Post-exposure** |
| **2019** | Q12 | 2018 | non-exposed | 36 (12+12+12) | 78 (12+12+12+18+24) |
|  | Q79 | 2018 |  | 36 (12+12+12) | 78 (12+12+12+18+24) |
|  | Q64.2 | 2017 |  | 36 (12+12+12) | 48 (12+12+6+24) |
|  | Q45 | 2018 |  | 36 (12+12+12) | 78 (12+12+12+18+24) |
|  | Q60 | 2019 | low-exposed | 36 (12+12+12) | 78 (12+12+12+18+24) |
|  | Q39 | 2018 |  | 36 (12+12+12) | 78 (12+12+12+18+24) |
|  | Q4 | 2018 |  | 36 (12+12+12) | 78 (12+12+12+18+24) |
|  | Q5 | 2017 |  | 36 (12+12+12) | 66 (12+12+18+24) |
|  | Q71 | 2019 |  | 24 (12+12) | 78 (12+12+12+18+24) |
|  | Q88 | 2018 |  | 24 (12+12) | 78 (12+12+12+18+24) |
| **2020** | Q12 | 2018 | non-exposed | 60 (24+24+12) | 60 (24+12+12+12) |
|  | Q79 | 2018 |  | 60 (24+24+12) | 60 (24+12+12+12) |
|  | Q64.2 | 2017 |  | 60 (24+24+12) | 48 (24+12+12) |
|  | Q45 | 2018 | high-exposed | 60 (24+24+12) | 60 (24+12+12+12) |
|  | Q15 | 2020 |  | 60 (24+24+12) | 60 (24+12+12+12) |
|  | Q65 | 2020 |  | 60 (24+24+12) | 60 (24+12+12+12) |
|  | Q7 | 2020 |  | 60 (24+24+12) | 60 (24+12+12+12) |
|  | Q71 | 2019 | high-boosted | 60 (24+24+12) | 60 (24+12+12+12) |
|  | Q88 | 2018 |  | 60 (24+24+12) | 60 (24+12+12+12) |

The sample sizes for larvae inoculated with *Melissococcus plutonius* bacteria and for non-inoculated control larvae during each rearing period were the same. Boosted queens were exposed for a second time to a high dose of *M. plutonius*, one year after their first exposure with a low dose in 2019.
