# Supplementary material for: Lack of evidence for trans-generational immune priming against the honey bee pathogen Melissococcus plutonius
Source: PLoS One. 2022 May 9;17(5):e0268142. doi: 10.1371/journal.pone.0268142 (PMC9084521; doi:10.1371/journal.pone.0268142)
Supplement: S2 Table — (DOCX) [file pone.0268142.s002.docx]

**S2 Table. Number of larvae per queen collected from EFB-diseased colonies (i.e. naturally exposed to *M. plutonius*) or from healthy colonies (i.e. non-exposed to *M. plutonius*).**

|  |  |  | **Larval inoculation**  **(number of larvae per replicate)** | |
| --- | --- | --- | --- | --- |
| **Year** | **Queen** | **Queen group** | **Infected** | **Uninfected** |
| **2021** | M10 | non-exposed | 36 (12+12+12) | 36 (12+12+12) |
|  | M11 |  | 48 (12+12+12+12) | 48 (12+12+12+12) |
|  | M16 |  | 36 (12+12+12) | 36 (12+12+12) |
|  | M30 |  | 48 (12+12+12+12) | 48 (12+12+12+12) |
|  | M62 |  | 48 (12+12+12+12) | 47 (12+12+11+12) |
|  | M67 |  | 36 (12+12+12) | 36 (12+12+12) |
|  | M72 |  | 48 (12+12+12+12) | 48 (12+12+12+12) |
|  | M86 |  | 48 (12+12+12+12) | 48 (12+12+12+12) |
|  | M95 |  | 36 (12+12+12) | 36 (12+12+12) |
|  | M46 |  | 48 (12+12+12+12) | 48 (12+12+12+12) |
|  | M83 | exposed | 48 (12+12+12+12) | 48 (12+12+12+12) |
|  | M71 |  | 36 (12+12+12) | 35 (12+11+12) |
|  | M77 |  | 48 (12+12+12+12) | 48 (12+12+12+12) |
|  | M6 |  | 36 (12+12+12) | 36 (12+12+12) |
|  | M82 |  | 32 (12+12+8) | 32 (12+12+8) |
|  | M81 |  | 48 (12+12+12+12) | 48 (12+12+12+12) |
|  | M84 |  | 48 (12+12+12+12) | 48 (12+12+12+12) |
|  | M76 |  | 48 (12+12+12+12) | 48 (12+12+12+12) |
|  | M66 |  | 48 (12+12+12+12) | 48 (12+12+12+12) |
|  | M4 |  | 36 (12+12+12) | 36 (12+12+12) |
