# Supplementary material for: Lack of evidence for trans-generational immune priming against the honey bee pathogen Melissococcus plutonius
Source: PLoS One. 2022 May 9;17(5):e0268142. doi: 10.1371/journal.pone.0268142 (PMC9084521; doi:10.1371/journal.pone.0268142)
Supplement: S3 Table — (DOCX) [file pone.0268142.s003.docx]

**S3 Table. Composition of the diets used for larval rearing, according to feeding day.**

|  | **Day 1** | **Day 3** | **Day 4** | **Day 5** | **Day 6** |
| --- | --- | --- | --- | --- | --- |
| **Diet** | **A** | **B** | **C** | **C** | **C** |
| Volume per larva (µl) | 20 | 20 | 30 | 40 | 50 |
| Royal jelly (g) | 47.6 | 47.0 | 46.6 | 46.6 | 46.6 |
| Yeast extract (g) | 1 | 1.4 | 1.9 | 1.9 | 1.9 |
| D-glucose (g) | 5.7 | 7.0 | 8.4 | 8.4 | 8.4 |
| D-fructose (g) | 5.7 | 7.0 | 8.4 | 8.4 | 8.4 |
| MilliQ H2O (g) | 40.0 | 37.6 | 34.7 | 34.7 | 34.7 |
| Total (g) | 100 | 100 | 100 | 100 | 100 |
