# Supplementary material for: Lack of evidence for trans-generational immune priming against the honey bee pathogen Melissococcus plutonius
Source: PLoS One. 2022 May 9;17(5):e0268142. doi: 10.1371/journal.pone.0268142 (PMC9084521; doi:10.1371/journal.pone.0268142)
Supplement: S1 File — (DOCX) [file pone.0268142.s004.docx]

**S1-4 Materials**

**SM 1. PCR analysis and confirmation of *Melissococcus* *plutonius* presence and absence in test colonies**

To confirm the absence of *M. plutonius* in test colonies, approximately ten grams of adult workers from three colonies were sampled and pooled for analysis by qualitative real-time PCR using a system for *M. plutonius* detection (Dainat et al., 2018). To verify the presence of the bacteria in symptomatic colonies in the field, the same number of workers was analyzed for each colony individually. Upon field collection, the workers were frozen at -80°C until DNA extraction (Roetschi et al., 2008). Prior to DNA extraction, the workers were manually ground in an extraction bag with mesh (BIOREBA) filled with 15 ml TN Buffer (0.4M NaCl, 10mM Tris, pH=7). To the 200 µl of collected sample homogenate, 200 µl of a lysozyme solution (20 mg lysozyme/ml in: 20mM Tris/HCL, 2mM EDTA, 1% Triton X-100, pH=8) were added and incubated 1 hour at 37°C. Samples were then prepared according to the NucleoSpin Tissue kit from Macherey-Nagel following the user manual (Standard protocol). The qPCR was set up in a 12 µl volume, with Master Mix (2X) Universal (PROBE FAST qPCR Kit from Bioline) containing 400nM of each forward and reverse primers, 200nM of each probe and 2 µl DNA-extract. The primers and probes used are listed in Table 1. The actin gene of *A. mellifera* was amplified to evaluate the amplification ability of the extracted DNA as a control. In addition, positive and negative controls were run with, instead of sample DNA-extract, 2 µl pure *M. plutonius* extract, and 2 µl milliQ water respectively. The DNA amplifications were performed with an Illumina thermocycler (Eco^TM^ Real-Time PCR System). The cycling program was applied as follows: initial denaturation step at 95°C for 3 min, followed by 45 cycles of 3 s at 95°C and 30 s at 60°C. The limit of detection (LOD) at a quantification cycle (Cq) value of 30 for *M. plutonius* was used for the data analysis (Dainat et al., 2018). Samples were run in duplicate and their Cq value averaged (Cq mean). The results are presented in Table 2. All samples were positive for *A. mellifera* DNA and contained no or residual DNA of *M. plutonius* bacteria (Table 2). Exposure of the queens to low and high doses of viable *M. plutonius* cells did not lead to bacterial multiplication in the free-living colonies.

**Table 1. Primer probes and gene sequences used for qPCR analysis^1^.**

| Target organism | Gene | Primer/Probe | Oligonucleotide sequence (5’-3’) |
| --- | --- | --- | --- |
| *M. plutonius* | napA | MP-F | GAC CTG TTT AGC TAT TAT CAC TA |
|  |  | MP-R | CAC CTA CAA TGA ATG ATT CAT TC |
|  |  | MP-Probe | FAM - TCC GCC TAA GCT ACC ACC TAA GAA C |

^1^designed by Dainat et al. (2018).

**Table 2. Averaged quantification cycle (Cq mean) values obtained by** **qPCR for *Melissococcus plutonius* from workers sampled in symptomatic colonies upon queen collection.**

| **Year** | **Queen** | ***M. plutonius*** |
| --- | --- | --- |
| **2021** | M83 | 24.53 |
|  | M71 | 20.19 |
|  | M77 | 33.47 |
|  | M6 | 25.64 |
|  | M82 | 36.10 |
|  | M81 | 34.53 |
|  | M84 | 24.66 |
|  | M76 | 25.21 |
|  | M66 | 25.48 |
|  | M4 | 35.81 |

The limit of detection (LOD) for *M. plutonius* is set to 30 Cq mean values. Cq mean values are in green when representing a positive signal (Cq mean<LOD), and in yellow for residual (Cq mean>LOD) signals after 45 DNA-amplification cycles.

**SM 2. Intended and effective concentrations and doses in *Melissococcus plutonius* inocula.**

Targeted concentration of *Melissococcus plutonius* in CFUs per larva and mean ± standard error CFUs per larva measured after plating the inocula used for infection assays.

|  |  | **Larval infection (CFUs/larva)** | | |
| --- | --- | --- | --- | --- |
| **Year** | **Rearing period** | **Targeted** | **Inoculated** | **Non-inoculated** |
| **2019** | pre-exposure | 2x10^5^ | 3.42x10^4^ ± 1.28x10^4^ | 0 ± 0 |
|  | post-exposure | 2x10^5^ | 2.86x10^4^ ± 5.50x10^3^ | 0 ± 0 |
| **2020** | pre-exposure | 2x10^5^ | 4.44x10^4^ ± 1.02x10^4^ | 0 ± 0 |
|  | post-exposure | 2x10^5^ | 3.36x10^4^ ± 1.38x10^4^ | 0 ± 0 |
| **2021** | post-exposure | 2x10^5^ | 1.79x10^4^ ± 1.77x10^4^ | 0 ± 0 |

Targeted dose of *Melissococcus plutonius* in CFUs per queen and mean CFUs per queen measured after plating the inocula used for exposure. Boosted queens were exposed a second time to high dose of *M. plutonius*, one year after low exposure in 2019.

|  |  |  |  |
| --- | --- | --- | --- |
| **Year** | **Queen group** | **Targeted CFUs/queen** | **CFUs/queen** |
| **2019** | non-exposed | 0 | 0 |
|  | low-exposed | 5x10^5^ | 1.4x10^5^ |
| **2020** | non-exposed | 0 | 0 |
|  | high-exposed | 1x10^7^ | 9.6x10^6^ |
|  | high-boosted | 1x10^7^ | 9.6x10^6^ |

**SM 3. Script and output of Cox mixed-effects model of brood survival in 2019.**

> M2019 <- coxme(Surv(time, death) ~ queen_group * larva_treatment + (1|queen_identity/replicate),

+ ties = "breslow", data=X2019)

> summary(M2019)

Cox mixed-effects model fit by maximum likelihood

Data: X2019

events, n = 907, 2148

Iterations= 20 124

NULL Integrated Fitted

Log-likelihood -6768.707 -6374.463 -6324.699

Chisq df p AIC BIC

Integrated loglik 788.49 9.00 0 770.49 727.20

Penalized loglik 888.02 44.91 0 798.20 582.18

Model: Surv(time, death) ~ queen_group * larva_treatment + (1 | queen/serie)

Fixed coefficients

coef exp(coef) se(coef) z p

queen_group1 0.8909279 2.4373902 0.4381549 2.03 4.2e-02

queen_group2 1.1307888 3.0980995 0.3991209 2.83 4.6e-03

quenn_group3 0.9601925 2.6121993 0.4064460 2.36 1.8e-02

larva_treatment1 2.9988389 20.0622300 0.3681918 8.14 3.3e-16

queen_group1:larva_treatment1 -0.7995176 0.4495458 0.4273267 -1.87 6.1e-02

queen_group2:larva_treatment1 -0.9932342 0.3703769 0.4030399 -2.46 1.4e-02

queen_group3:larva_treatment1 -0.9489960 0.3871295 0.3928549 -2.42 1.6e-02

Random effects

Group Variable Std Dev Variance

queen_identity/replicate (Intercept) 0.29127878 0.08484333

queen_identity (Intercept) 0.18361333 0.03371385

> A) summary(emmeans(M2019, pairwise ~ larva_treatment, type = "response"))

NOTE: Results may be misleading due to involvement in interactions

$emmeans

larva_treatment response SE df asymp.LCL asymp.UCL

non inoculated 0.297 0.0236 Inf 0.254 0.347

inoculated 3.001 0.1641 Inf 2.696 3.340

Results are averaged over the levels of: q_group

Confidence level used: 0.95

Intervals are back-transformed from the log scale

$contrasts

contrast ratio SE df null z.ratio p.value

non inoculated / inoculated 0.0989 0.0119 Inf 1 -19.217 <.0001

Results are averaged over the levels of: queen_group

Tests are performed on the log scale

> B) summary(emmeans(M2019, pairwise ~ queen_group, type = "response"))

NOTE: Results may be misleading due to involvement in interactions

$emmeans

queen_group response SE df asymp.LCL asymp.UCL

queen non exposed pre 0.631 0.1239 Inf 0.429 0.927

queen exposed pre 1.031 0.1336 Inf 0.800 1.329

queen non exposed post 1.190 0.1410 Inf 0.943 1.501

queen exposed post 1.025 0.0851 Inf 0.872 1.207

Results are averaged over the levels of: larva_treatment

Confidence level used: 0.95

Intervals are back-transformed from the log scale

$contrasts

contrast ratio SE df null z.ratio p.value

queen non exposed pre / queen exposed pre 0.612 0.164 Inf 1 -1.827 0.2603

queen non exposed pre / queen non exposed post 0.530 0.121 Inf 1 -2.769 0.0288

queen non exposed pre / queen exposed post 0.615 0.154 Inf 1 -1.937 0.2124

queen exposed pre / queen non exposed post 0.867 0.179 Inf 1 -0.692 0.9004

queen exposed pre / queen exposed post 1.005 0.159 Inf 1 0.035 1.0000

queen non exposed post/ queen exposed post 1.160 0.212 Inf 1 0.813 0.8484

Results are averaged over the levels of: larva_treatment

P value adjustment: tukey method for comparing a family of 4 estimates

Tests are performed on the log scale

> C) summary(emmeans(M2019, pairwise ~ queen_group * larva_treatment, type = "response"))

$emmeans

queen_group larva_treatment response SE df asymp.LCL asymp.UCL

queen non exposed pre non inoculated 0.141 0.0493 Inf 0.071 0.280

queen exposed pre non inoculated 0.343 0.0706 Inf 0.230 0.514

queen non exposed post non inoculated 0.436 0.0721 Inf 0.316 0.603

queen exposed post non inoculated 0.368 0.0455 Inf 0.289 0.469

queen non exposed pre inoculated 2.826 0.4253 Inf 2.104 3.796

queen exposed pre inoculated 3.097 0.3798 Inf 2.435 3.938

queen non exposed post inoculated 3.243 0.3900 Inf 2.562 4.105

queen exposed post inoculated 2.858 0.2568 Inf 2.396 3.408

Confidence level used: 0.95

Intervals are back-transformed from the log scale

$contrasts

contrast ratio SE df null z.ratio p.value

1) queen non exposed pre - larva non inoculated / queen non exposed pre - larva inoculated

0 0 / 0 1 0.0498 0.0184 Inf 1 -8.145 <.0001

2) queen exposed pre - larva non inoculated / queen exposed pre - larva inoculated

1 0 / 1 1 0.1109 0.0242 Inf 1 -10.096 <.0001

3) queen non exposed post - larva non inoculated / queen non exposed post - larva inoculated

2 0 / 2 1 0.1346 0.0222 Inf 1 -12.143 <.0001

4) queen exposed post - larva non inoculated / queen exposed post - larva inoculated

3 0 / 3 1 0.1288 0.0178 Inf 1 -14.817 <.0001

5) queen non exposed pre - larva non inoculated / queen exposed pre - larva non inoculated

0 0 / 1 0 0.4103 0.1798 Inf 1 -2.033 0.4591

6) queen non exposed pre - larva non inoculated / queen non exposed post - larva non inoculated

0 0 / 2 0 0.3228 0.1288 Inf 1 -2.833 0.0869

7) queen exposed pre - larva non inoculated / queen exposed post - larva non inoculated

1 0 / 3 0 0.9331 0.2368 Inf 1 -0.273 1.0000

8) queen non exposed pre - larva inoculated / queen exposed pre - larva inoculated

0 1 / 1 1 0.9126 0.1910 Inf 1 -0.437 0.9999

9) queen non exposed pre - larva inoculated / queen non exposed post - larva inoculated

0 1 / 2 1 0.8715 0.1428 Inf 1 -0.840 0.9908

10) queen exposed pre - larva inoculated / queen exposed post - larva inoculated

1 1 / 3 1 1.0835 0.1487 Inf 1 0.585 0.9991

11) queen non exposed post - larva inoculated / queen exposed post - larva inoculated

2 1 / 3 1 1.1347 0.1962 Inf 1 0.731 0.9961

12) queen non exposed pre - larva non inoculated / queen exposed post - larva non inoculated

0 0 / 3 0 0.3828 0.1556 Inf 1 -2.362 0.2601

13) queen non exposed pre - larva non inoculated / queen exposed pre - larva inoculated

0 0 / 1 1 0.0455 0.0181 Inf 1 -7.747 <.0001

14) queen non exposed pre - larva non inoculated / queen non exposed post - larva inoculated

0 0 / 2 1 0.0434 0.0164 Inf 1 -8.319 <.0001

15) queen non exposed pre - larva non inoculated / queen exposed post - larva inoculated

0 0 / 3 1 0.0493 0.0193 Inf 1 -7.697 <.0001

16) queen exposed pre - larva non inoculated / queen non exposed post - larva non inoculated

1 0 / 2 0 0.7867 0.2315 Inf 1 -0.815 0.9923

17) queen exposed pre - larva non inoculated / queen non exposed pre - larva inoculated

1 0 / 0 1 0.1215 0.0337 Inf 1 -7.592 <.0001

18) queen exposed pre - larva non inoculated / queen non exposed post - larva inoculated

1 0 / 2 1 0.1059 0.0279 Inf 1 -8.522 <.0001

19) queen exposed pre - larva non inoculated / queen exposed post - larva inoculated

1 0 / 3 1 0.1201 0.0274 Inf 1 -9.281 <.0001

20) queen non exposed post - larva non inoculated / queen exposed post - larva non inoculated

2 0 / 3 0 1.1860 0.2901 Inf 1 0.698 0.9971

21) queen non exposed post - larva non inoculated / queen non exposed pre - larva inoculated

2 0 / 0 1 0.1544 0.0325 Inf 1 -8.866 <.0001

22) queen non exposed post - larva non inoculated / queen exposed pre - larva inoculated

2 0 / 1 1 0.1409 0.0327 Inf 1 -8.457 <.0001

23) queen non exposed post - larva non inoculated / queen exposed post - larva inoculated

2 0 / 3 1 0.1527 0.0333 Inf 1 -8.624 <.0001

24) queen exposed post - larva non inoculated / queen non exposed pre - larva inoculated

3 0 / 0 1 0.1302 0.0292 Inf 1 -9.089 <.0001

25) queen exposed post - larva non inoculated / queen exposed pre - larva inoculated

3 0 / 1 1 0.1188 0.0211 Inf 1 -11.997 <.0001

26) queen exposed post - larva non inoculated / queen non exposed post - larva inoculated

3 0 / 2 1 0.1135 0.0234 Inf 1 -10.540 <.0001

27) queen non exposed pre - larva inoculated / queen exposed post - larva inoculated

0 1 / 3 1 0.9889 0.1919 Inf 1 -0.058 1.0000

28) queen exposed pre - larva inoculated / queen non exposed post - larva inoculated

1 1 / 2 1 0.9549 0.1813 Inf 1 -0.243 1.0000

P value adjustment: tukey method for comparing a family of 8 estimates

Tests are performed on the log scale

**SM 4. Script and output of Cox mixed-effects model of brood survival in 2020.**

> M2020 <- coxme(Surv(time, death) ~ queen_group * larva_treatment + (1|queen_identity/repicate),

+ ties = "breslow", data=X2020)

> summary(M2020)

Cox mixed-effects model fit by maximum likelihood

Data: X2020

events, n = 1172, 2136

Iterations= 21 130

NULL Integrated Fitted

Log-likelihood -8654.595 -8094.959 -8046.411

Chisq df p AIC BIC

Integrated loglik 1119.27 13.00 0 1093.27 1027.41

Penalized loglik 1216.37 45.27 0 1125.83 896.48

Model: Surv(time, death) ~ queen_treatment.f * larva_treatment.f + (1 | queen.f/serie.f)

Fixed coefficients

coef exp(coef) se(coef) z p

queen_group1 0.42023473 1.5223188 0.2656328 1.58 0.11000

queen_group2 0.83655620 2.3084036 0.2912898 2.87 0.00410

queen_group3 -0.17940369 0.8357684 0.3047837 -0.59 0.56000

queen_group4 -0.04928966 0.9519054 0.2794551 -0.18 0.86000

queen_group5 0.70670983 2.0273101 0.2982689 2.37 0.01800

larva_treatment1 2.40191957 11.0443565 0.2030323 11.83 0.00000

queen_group1:larva_treatment1 -0.26103513 0.7702539 0.2511834 -1.04 0.30000

queen_group2:larva_treatment1 -0.96494327 0.3810048 0.2733609 -3.53 0.00042

queen_group3:larva_treatment1 0.25076220 1.2850045 0.2955781 0.85 0.40000

queen_group4:larva_treatment1 0.28440269 1.3289680 0.2695868 1.05 0.29000

queen_group5:larva_treatment1 -0.65574252 0.5190565 0.2859517 -2.29 0.02200

Random effects

Group Variable Std Dev Variance

queen_identity/replicate (Intercept) 0.3029822821 **0.0917982633**

queen_identity (Intercept) 0.0140794673 **0.0001982314**

> A) summary(emmeans(M2020, pairwise ~ larva_treatment, type = "response"))

NOTE: Results may be misleading due to involvement in interactions

$emmeans

larva_treatment response SE df asymp.LCL asymp.UCL

non inoculated 0.344 0.0149 Inf 0.316 0.375

inoculated 3.036 0.1325 Inf 2.788 3.308

Results are averaged over the levels of: queen_group

Confidence level used: 0.95

Intervals are back-transformed from the log scale

$contrasts

contrast ratio SE df null z.ratio p.value

larva non inoculated / larva inoculated 0.113 0.00913 Inf 1 -27.029 <.0001

Results are averaged over the levels of: queen_group

Tests are performed on the log scale

> B) summary(emmeans(M2020, pairwise ~ queen_group, type = "response"))

NOTE: Results may be misleading due to involvement in interactions

$emmeans

queen_group response SE df asymp.LCL asymp.UCL

queen non exposed pre 0.856 0.1111 Inf 0.664 1.10

queen exposed pre 1.144 0.1173 Inf 0.936 1.40

queen non exposed post 1.220 0.1811 Inf 0.912 1.63

queen exposed post 0.811 0.1031 Inf 0.633 1.04

queen boosted pre 0.940 0.0975 Inf 0.767 1.15

queen boosted post 1.251 0.1867 Inf 0.934 1.68

Results are averaged over the levels of: larva_treatment

Confidence level used: 0.95

Intervals are back-transformed from the log scale

$contrasts

contrast ratio SE df null z.ratio p.value

queen non exposed pre / queen exposed pre 0.748 0.139 Inf 1 -1.564 0.6224

queen non exposed pre / queen non exposed post 0.702 0.149 Inf 1 -1.665 0.5550

queen non exposed pre / queen exposed post 1.056 0.212 Inf 1 0.269 0.9998

queen non exposed pre / queen boosted pre 0.911 0.170 Inf 1 -0.499 0.9962

queen non exposed pre / queen boosted post 0.685 0.145 Inf 1 -1.791 0.4712

queen exposed pre / queen non exposed post 0.938 0.183 Inf 1 -0.330 0.9995

queen exposed pre / queen exposed post 1.410 0.257 Inf 1 1.888 0.4096

queen exposed pre / queen boosted pre 1.218 0.201 Inf 1 1.191 0.8412

queen exposed pre / queen boosted post 0.915 0.177 Inf 1 -0.460 0.9974

queen non exposed post / queen exposed post 1.504 0.316 Inf 1 1.944 0.3754

queen non exposed post / queen boosted pre 1.298 0.254 Inf 1 1.334 0.7660

queen non exposed post / queen boosted post 0.976 0.214 Inf 1 -0.113 1.0000

queen exposed post / queen boosted pre 0.863 0.159 Inf 1 -0.800 0.9676

queen exposed post / queen boosted post 0.649 0.136 Inf 1 -2.067 0.3046

queen boosted pre / queen boosted post 0.751 0.147 Inf 1 -1.464 0.6871

Results are averaged over the levels of: larva_treatment

P value adjustment: tukey method for comparing a family of 6 estimates

Tests are performed on the log scale

> C) summary(emmeans(M2020, pairwise ~ queen_group * larva_treatment, type = "response"))

$emmeans

queen_group larva_treatment response SE df asymp.LCL asymp.UCL

queen non exposed pre larva non inoculated 0.258 0.0508 Inf 0.175 0.379

queen exposed pre larva non inoculated 0.392 0.0571 Inf 0.295 0.522

queen non exposed post larva non inoculated 0.595 0.1140 Inf 0.409 0.866

queen exposed post larva non inoculated 0.215 0.0441 Inf 0.144 0.322

queen boosted pre larva non inoculated 0.245 0.0407 Inf 0.177 0.340

queen boosted post larva non inoculated 0.522 0.1062 Inf 0.351 0.778

queen non exposed pre larva inoculated 2.846 0.3534 Inf 2.231 3.630

queen exposed pre larva inoculated 3.337 0.3541 Inf 2.711 4.109

queen non exposed post larva inoculated 2.503 0.3920 Inf 1.842 3.403

queen exposed post larva inoculated 3.057 0.3614 Inf 2.424 3.854

queen boosted pre larva inoculated 3.601 0.3628 Inf 2.955 4.387

queen boosted post larva inoculated 2.995 0.4629 Inf 2.212 4.055

Confidence level used: 0.95

Intervals are back-transformed from the log scale

$contrasts

contrast ratio SE df null z.ratio p.value

1) queen non exposed pre - larva non inoculated / queen non exposed pre - larva inoculated

0.0905 0.0184 Inf 1 -11.830 <.0001

2) queen exposed pre - larva non inoculated / queen exposed pre - larva inoculated

0.1176 0.0178 Inf 1 -14.160 <.0001

3) queen non exposed post - larva non inoculated / queen non exposed post - larva inoculated

0.2376 0.0441 Inf 1 -7.745 <.0001

4) queen exposed post - larva non inoculated / queen exposed post - larva inoculated

0.0705 0.0153 Inf 1 -12.225 <.0001

5) queen boosted pre - larva non inoculated / queen boosted pre - larva inoculated

0.0681 0.0123 Inf 1 -14.908 <.0001

6) queen boosted post - larva non inoculated / queen boosted post - larva inoculated

0.1744 0.0355 Inf 1 -8.586 <.0001

7) queen non exposed pre - larva non inoculated / queen exposed pre - larva non inoculated

0.6569 0.1745 Inf 1 -1.582 0.9159

8) queen non exposed pre - larva inoculated / queen exposed pre - larva inoculated

0.8528 0.1467 Inf 1 -0.925 0.9989

9) queen non exposed pre - larva non inoculated / queen boosted pre - larva non inoculated

1.0505 0.2936 Inf 1 0.176 1.0000

10) queen non exposed pre - larva inoculated / queen boosted pre - larva inoculated

0.7905 0.1310 Inf 1 -1.418 0.9603

11) queen exposed pre - larva non inoculated / queen boosted pre - larva non inoculated

1.5992 0.3868 Inf 1 1.941 0.7331

12) queen exposed pre - larva inoculated / queen boosted pre - larva inoculated

0.9269 0.1411 Inf 1 -0.499 1.0000

13) queen non exposed post - larva non inoculated / queen exposed post - larva non inoculated

2.7620 0.8186 Inf 1 3.428 0.0300

14) queen non exposed post - larva inoculated / queen exposed post - larva inoculated

0.8189 0.1651 Inf 1 -0.991 0.9979

15) queen non exposed post - larva non inoculated / queen boosted post - larva non inoculated

1.1387 0.3292 Inf 1 0.449 1.0000

16) queen non exposed post - larva inoculated / queen boosted post - larva inoculated

0.8358 0.1875 Inf 1 -0.799 0.9997

17) queen exposed post - larva non inoculated / queen boosted post - larva non inoculated

0.4123 0.1252 Inf 1 -2.918 0.1343

18) queen exposed post - larva inoculated / queen boosted post - larva inoculated

1.0206 0.2023 Inf 1 0.103 1.0000

19) queen non exposed pre - larva non inoculated / queen non exposed post - larva non inoculated

0.4332 0.1262 Inf 1 -2.872 0.1509

20) queen exposed pre - larva non inoculated / queen exposed post - larva non inoculated

1.8215 0.4939 Inf 1 2.211 0.5411

21) queen boosted pre - larva non inoculated / queen boosted post - larva non inoculated

0.4695 0.1306 Inf 1 -2.719 0.2168

22) queen non exposed pre - larva inoculated / queen non exposed post - larva inoculated

1.1370 0.2356 Inf 1 0.620 1.0000

23) queen exposed pre - larva inoculated / queen exposed post - larva inoculated

1.0918 0.1802 Inf 1 0.532 1.0000

24) queen boosted pre - larva inoculated / queen boosted post - larva inoculated

1.2022 0.2261 Inf 1 0.979 0.9981

25) queen non exposed pre - larva non inoculated / queen exposed post - larva non inoculated

1.1965 0.3647 Inf 1 0.589 1.0000

26) queen non exposed pre - larva non inoculated / queen boosted post - larva non inoculated

0.4933 0.1471 Inf 1 -2.369 0.4271

27) queen non exposed pre - larva non inoculated / queen exposed pre - larva inoculated

0.0772 0.0186 Inf 1 -10.609 <.0001

28) queen non exposed pre - larva non inoculated / queen non exposed post - larva inoculated

0.1029 0.0275 Inf 1 -8.498 <.0001

29) queen non exposed pre - larva non inoculated / queen exposed post - larva inoculated

0.0843 0.0206 Inf 1 -10.108 <.0001

30) queen non exposed pre - larva non inoculated / queen boosted pre - larva inoculated

0.0716 0.0170 Inf 1 -11.130 <.0001

31) queen non exposed pre - larva non inoculated / queen boosted post - larva inoculated

0.0860 0.0228 Inf 1 -9.274 <.0001

32) queen exposed pre - larva non inoculated / queen non exposed post - larva non inoculated

0.6595 0.1686 Inf 1 -1.629 0.8988

33) queen exposed pre - larva non inoculated / queen boosted post - larva non inoculated

0.7509 0.1979 Inf 1 -1.087 0.9953

34) queen exposed pre - larva non inoculated / queen non exposed pre - larva inoculated

0.1378 0.0285 Inf 1 -9.571 <.0001

35) queen exposed pre - larva non inoculated / queen non exposed post - larva inoculated

0.1567 0.0358 Inf 1 -8.122 <.0001

36) queen exposed pre - larva non inoculated / queen exposed post - larva inoculated

0.1283 0.0258 Inf 1 -10.202 <.0001

37) queen exposed pre - larva non inoculated / queen boosted pre - larva inoculated

0.1090 0.0208 Inf 1 -11.598 <.0001

38) queen exposed pre - larva non inoculated / queen boosted post - larva inoculated

0.1310 0.0294 Inf 1 -9.050 <.0001

39) queen non exposed post - larva non inoculated / queen boosted pre - larva non inoculated

2.4250 0.6548 Inf 1 3.281 0.0481

40) queen non exposed post - larva non inoculated / queen non exposed pre - larva inoculated

0.2090 0.0500 Inf 1 -6.549 <.0001

41) queen non exposed post - non inoculated / queen exposed pre - larva inoculated

0.1783 0.0410 Inf 1 -7.489 <.0001

42) queen non exposed post - larva non inoculated / queen exposed post - larva inoculated

0.1946 0.0456 Inf 1 -6.993 <.0001

43) queen non exposed post - larva non inoculated / queen boosted pre - larva inoculated

0.1652 0.0373 Inf 1 -7.979 <.0001

44) queen non exposed post - larva non inoculated / queen boosted post - larva inoculated

0.1986 0.0505 Inf 1 -6.362 <.0001

45) queen exposed post - larva non inoculated / queen boosted pre - larva non inoculated

0.8780 0.2504 Inf 1 -0.456 1.0000

46) queen exposed post - larva non inoculated / queen non exposed pre - larva inoculated

0.0757 0.0193 Inf 1 -10.105 <.0001

47) queen exposed post - larva non inoculated / queen exposed pre - larva inoculated

0.0645 0.0160 Inf 1 -11.070 <.0001

48) queen exposed post - larva non inoculated / queen non exposed post - larva inoculated

0.0860 0.0235 Inf 1 -8.981 <.0001

49) queen exposed post - larva non inoculated / queen boosted pre - larva inoculated

0.0598 0.0146 Inf 1 -11.555 <.0001

50) queen exposed post - larva non inoculated / queen boosted post - larva inoculated

0.0719 0.0195 Inf 1 -9.726 <.0001

51) queen boosted pre - larva non inoculated / queen non exposed pre - larva inoculated

0.0862 0.0194 Inf 1 -10.912 <.0001

52) queen boosted pre - larva non inoculated / queen exposed pre - larva inoculated

0.0735 0.0158 Inf 1 -12.137 <.0001

53) queen boosted pre - larva non inoculated / queen non exposed post - larva inoculated

0.0980 0.0239 Inf 1 -9.509 <.0001

54) queen boosted pre - larva non inoculated / queen exposed post - larva inoculated

0.0803 0.0176 Inf 1 -11.474 <.0001

55) queen boosted pre - larva non inoculated / queen boosted post - larva inoculated

0.0819 0.0198 Inf 1 -10.359 <.0001

56) queen boosted post - larva non inoculated / queen non exposed pre - larva inoculated

0.1836 0.0454 Inf 1 -6.853 <.0001

57) queen boosted post - larva non inoculated / queen exposed pre - larva inoculated

0.1565 0.0374 Inf 1 -7.762 <.0001

58) queen boosted post - larva non inoculated / queen non exposed post - larva inoculated

0.2087 0.0553 Inf 1 -5.913 <.0001

59) queen boosted post - larva non inoculated / queen exposed post - larva inoculated

0.1709 0.0416 Inf 1 -7.267 <.0001

60) queen boosted post - larva non inoculated / queen boosted pre - larva inoculated

0.1451 0.0341 Inf 1 -8.210 <.0001

61) queen non exposed pre - larva inoculated / queen exposed post - larva inoculated

0.9311 0.1647 Inf 1 -0.403 1.0000

62) queen non exposed pre - larva inoculated / queen boosted post - larva inoculated

0.9503 0.1933 Inf 1 -0.251 1.0000

63) queen exposed pre - larva inoculated / queen non exposed post - larva inoculated

1.3332 0.2625 Inf 1 1.461 0.9510

64) queen exposed pre - larva inoculated / queen boosted post - larva inoculated

1.1143 0.2148 Inf 1 0.561 1.0000

65) queen non exposed post - larva inoculated / queen boosted pre - larva inoculated

0.6952 0.1331 Inf 1 -1.898 0.7608

66) queen exposed post - larva inoculated / queen boosted pre - larva inoculated

0.8490 0.1353 Inf 1 -1.028 0.9971

P value adjustment: tukey method for comparing a family of 12 estimates

Tests are performed on the log scale
